# Excess weight among colorectal cancer survivors: target for intervention

**DOI:** 10.1007/s00535-012-0567-2

**Published:** 2012-03-17

**Authors:** I. Soerjomataram, M. S. Y. Thong, I. J. Korfage, S. Polinder, A. van der Heide, E. de Vries, J. A. Rietjens, S. J. Otto, L. V. van de Poll-Franse

**Affiliations:** 1 Department of Public Health, Erasmus MC, Rotterdam, The Netherlands; 2 Comprehensive Cancer Centre South (CCCS), Eindhoven Cancer Registry, Eindhoven, The Netherlands; 3 CoRPS–Center of Research on Psychology in Somatic Diseases, Department of Medical Psychology, Tilburg University, Tilburg, The Netherlands; 4 Present Address: Cancer Information Section, International Agency for Research on Cancer, Lyon, France

**Keywords:** Alcohol, Colorectal cancer, Body mass index, Smoking, Survivors

## Abstract

**Background:**

Healthy lifestyle might improve outcome among colorectal cancer (CRC) survivors. In this study we investigated the proportion of survivors who meet recommended lifestyle and weight guidelines and compared this to the general population. Factors that predict current behaviour were also assessed.

**Method:**

A random sample of CRC survivors diagnosed between 1998 and 2007 were surveyed. Percentages of current smokers, alcohol consumers, excess weight and clustering of these variables were calculated. Using logistic regression we assessed demographical and clinical factors that predict current lifestyle and excess weight.

**Results:**

We included 1349 (74% response rate) survivors in this study of whom only 8 and 16% of male and female survivors met the recommended lifestyle and body weight. Among male survivors up to 10% had at least two unhealthy lifestyle factors and among women, up to 19%. The proportion of smokers and those who had ever consumed alcohol was lower compared to the general population (13 vs. 31%, 82 vs. 86% respectively), but excess weight (BMI at least 25 kg/m^2^) was more prevalent among survivors (69 vs. 53% respectively). Having received chemotherapy was significantly associated with being overweight (adjusted odd ratio 1.5, 95% confidence interval 1.05–2.3). Younger patients, male gender and survivors of lower socioeconomic status were more likely to show non-compliance to healthy lifestyle recommendations.

**Conclusion:**

The observed clustering of unhealthy lifestyle warrants interventions targeting multiple behaviours simultaneously. Reducing excess weight should be one of the most important targets of interventions, particularly for males, those who had chemotherapy and survivors of lower socioeconomic status.

## Introduction

Colorectal cancer (CRC) is one of the most common cancers in developed countries, and its incidence continues to rise. In 2008, 12000 new patients were diagnosed with CRC in the Netherlands as compared to 7000 cases in 1998 [1]. On the other hand, mortality has decreased and survivorship has improved, leading to an increase in the prevalence of CRC survivors. The number of CRC survivors is predicted to be 67000 by 2015, almost double the observed number for the year 2000 (34000 survivors) [2].

Lifestyle is an important factor that modifies outcomes including survival and quality of life among CRC survivors [3–6]. However, in terms of the lifestyle behaviour clusters (physical activity, fruit and vegetable consumption, and smoking), only 5% of cancer survivors meet all healthy living recommendations [7].

In this study we aimed to describe the prevalence of healthy behaviours on the basis of a population-based sample of CRC survivors. At the same time we also assessed demographical and clinical factors that were related to the current opted lifestyle among survivors.

## Method

In 2009 postal questionnaires were sent to assess health status among a random sample of 1682 CRC survivors within a population-based cancer registry in the south of the Netherlands. This registry (Eindhoven Cancer Registry, ECR) records data of all newly diagnosed cancer cases within an area of 2.4 million inhabitants. For this study, all CRC patients recorded in the ECR database who were diagnosed with the cancer between 1998 and 2007 were eligible for participation. We randomly selected survivors using weights based on cancer site, sex and year of diagnosis. The weights were derived from the total distribution of CRC survivors in the ECR region. Survivors with shorter years of follow-up since diagnosis were oversampled for inclusion in future follow-up assessments. Medical specialists sent their (former) patients a letter to inform them about the study, with the questionnaire attached to it. If the questionnaire was not returned within 2 months, a reminder-letter with an additional copy of the questionnaire was sent. A more detailed explanation on the procedure of the data collection has been described elsewhere [8, 9]. Approval for this study was obtained from a local certified medical ethics committee.

Data on clinical characteristics (tumour stage at diagnosis [10], type of primary treatment (surgery, chemotherapy and radiotherapy) and comorbidity [11]) were obtained from the registry as well as data on age, sex and socioeconomic class (based on zip codes [12]). Major lifestyle behaviours were assessed using standardized questionnaires on smoking (non-smokers, ex-smokers and smokers) and alcohol consumption (non-drinkers, ex-drinkers and drinkers). In addition we also described body mass index (BMI) based on self-reported body height and weight. Normal weight was defined as BMI less than 25 kg/m^2^, overweight as BMI 25–29.9 kg/m^2^ and obesity as BMI at least 30 kg/m^2^. Reported lifestyle factors by age groups were retrieved from the national household survey of the Netherlands Statistics Office to serve as a reference for the general Dutch population [13] and to enable comparison with our cohort. This survey is performed annually on about 10000 persons using computer-assisted personal interviewing.

### Analysis

Analyses were based on 1349 survivors who responded (74% response rate) to the questionnaire and to one or more questions on lifestyle factors that we examined. We determined proportions of smoking and alcohol intake history, excess weight and clustering of one or more unhealthy behaviours or excess weight among the survivors. Statistical difference between the groups among the survivors was tested using Fisher’s exact test and we considered the difference to the null hypothesis to be significant if the* p* value was lower than 0.05. Finally, we performed a logistic regression analysis to estimate the relation between patients’ or clinical factors with current unhealthy behaviour or weight. Dependent factors (lifestyles and BMI) were each grouped into two categories (current smoking: yes or no; alcohol consumption: current consumption or not; excess weight (BMI at least 25 kg/m^2^): yes or no). In the analysis we included gender, age at diagnosis, socioeconomic factors, cancer stage, comorbidity at diagnosis and time since diagnosis. All statistical analyses were performed using Stata (version 11 for Windows, StataCorp, TX, USA).

## Results

Table 1 summarizes the characteristics of the CRC survivors included in this study as well as of those who did not respond to the questionnaire or had missing values for behaviour- or BMI-related questions. Non-responders were more likely to be older than 65 years, had cancer of the colon and had one or more comorbidity at time of diagnosis. The elderly was the largest group in our study, comprising 57% of the survivors included, who responded to the questionnaire and therefore generally they had other coexisting illness at the time of diagnosis (52%). More than half of the responders (58%) were diagnosed with CRC 2–5 years before the survey took place and were diagnosed with stage I or II CRC (28 and 38%, respectively).

**Table 1:** Characteristics of CRC survivors according to response

	Responders^a^ (number, %)	Non-responders (number, %)	*p* value
Age at diagnosis (years)			<0.001
25–44	38 (3)	12 (4)	
45–64	548 (41)	97 (29)	
≥65	763 (57)	225 (67)	
Site			0.021
Colon	892 (66)	243 (73)	
Rectum	457 (34)	91 (27)	
Stage			0.168
I	381 (28)	89 (27)	
II	517 (38)	146 (44)	
III	382 (28)	89 (27)	
IV	69 (5)	10 (3)	
Years since diagnosis			0.460
<2	163 (12)	40 (12)	
2–4.9	784 (58)	183 (55)	
≥5	402 (30)	111 (33)	
Socioeconomic status			0.508
I (lowest)	289 (21)	78 (23)	
II	522 (39)	138 (41)	
III (highest)	469 (35)	100 (30)	
Institutionalized	32 (2)	10 (3)	
Unknown	37 (3)	8 (2)	
Comorbidity at diagnosis			0.005
No	538 (40)	101 (30)	
Yes/at least one condition	705 (52)	204 (61)	
Unknown	106 (8)	29 (9)	
Total	1349	334	

^a^Responded to question on smoking status, alcohol consumption or body weight and height

Table 2 presents the distribution of lifestyle factors among the CRC survivors. In this table we included the same results as reported from the national household survey. Participants in these surveys are selected on the basis of a random sample of the population registered in the cancer registry (for the survivors) or the municipality (for the general population). Because of the difference in age pattern in the two background populations, i.e. older people among the survivors, comparison in Table 2 should be done with caution. Comparisons are presented by age groups to circumvent the bias that might be introduced because of the difference in the age distribution of the two populations. Compared to the general population, CRC survivors were less likely to be a current smoker or to ever have consumed alcohol. Thirteen per cent of male survivors currently smoke cigarettes as compared to 31% among the general population, whereas 8 and 23% females smoked among the survivors and the general population respectively. Similar observations were found by age groups. A significant higher proportion of smokers was observed for male and younger survivors. Excess weight was more common among CRC survivors than in the general Dutch population [64% (48% with BMI 25–29.9 kg/m^2^ and 16% with BMI at least 30 kg/m^2^) vs. 47% (35% with BMI 25–29.9 kg/m^2^ and 12% BMI at least 30 kg/m^2^) respectively]. Among the survivors, males had significantly higher BMI than females. However, unlike smoking and alcohol consumption, BMI did not significantly vary by age groups.

**Table 2:** Lifestyle factor prevalence among CRC survivors as compared to general population of the Netherlands in 2009

Lifestyles/characteristics	Survivors (%)	Population (%)
Current smokers^a^ by sex^b^		
Male	13	31
Female	8	23
Current smokers^a^ per age group (age at survey, years)^b^		
25–44	22	33
45–64	17	29
≥65	8	15
BMI^a^ (kg/m^2^) by sex^b^		
Male	25–29: 54	25–29: 41
	≥30: 14	≥30: 11
Female	25–29: 40	25–29: 30
	≥30: 18	≥30: 12
BMI^a^ (kg/m^2^) by age group (age at survey, years)		
25–44	25–29: 42	25–29: 31
	≥30: 11	≥30: 10
45–64	25–29: 43	25–29: 41
	≥30: 17	≥30: 14
≥65	25–29: 50	25–29: 42
	≥30: 15	≥30: 15
Abstainer (alcohol)^a^ by gender^b^		
Male	18	14
Female	50	26
Abstainer^a^ by age group (age at survey, year)^b^		
25–44	38	14
45–64	23	15
≥65	38	28

Out of 1349 responders, 1296 reported their weight and height (missing: 4%), 1294 reported their smoking status (missing: 4%) and 949 reported their alcohol intake (missing: 29%)

^a^Lifestyle factors including smoking status, alcohol consumption and BMI were categorised as follows: (1) current smoking: yes or no (includes lifelong non-smokers and ex-smokers), (2) alcohol consumption: abstainer and not (includes ex-consumer and current consumer) and (3) BMI 25–29.9 kg/m^2^ for overweight and BMI ≥ 30 kg/m^2^ for obese

^b^Exact Fisher test for comparison of proportions between subgroups among CRC survivors were significant with a *p* value less than 0.05

Table 3 shows the clustering between lifestyle factors and excess weight among the CRC survivors in our study. Among male and female survivors, only 8 and 18% respectively were non-smokers, alcohol abstainers and of normal weight. Male survivors had a stronger clustering of risk factors that include smoking compared to females (smoker and alcohol consumer 10 versus 4% or smoker and excess weight 8 versus 4% for male and female, respectively). On the other hand, more female survivors were, at the same time, overweight and alcohol consumers as compared to males (19 vs. 9% respectively).

**Table 3:** Clustering of lifestyle factors and excess weight among CRC survivors according to gender

	Male (%)	Female (%)	*p* value^a^
Currently smoke, consume alcohol and excess weight	5.9	1.9	0.000
Currently smoke and consume alcohol	9.8	3.6	0.000
Currently smoke and excess weight	7.8	4.3	0.037
Currently consume alcohol and excess weight	9.0	19.4	0.002
Non-smokers, alcohol abstainers and normal weight	7.8	17.5	0.000

Lifestyle factors including smoking status, alcohol consumption and BMI were categorised as follows: (1) current smoking: yes or no (includes lifelong non-smokers and ex-smokers), (2) alcohol consumption: yes and no (includes ex-consumer or never consumed alcohol) and (3) BMI 25–29.9 kg/m^2^ for overweight and BMI ≥ 30 kg/m^2^ for obese. Out of 1349 survivors included in the study 907 (67%) responded to all three questions (smoking, alcohol consumption and weight and height)

^a^Exact Fisher test on proportional difference between male and female survivors

Table 4 describes the relation between patient characteristics (at the time of diagnosis) and current behaviour or BMI. Female CRC patients were less likely than males to currently smoke [odds ratio (OR) 0.5, 95% confidence interval (95CI) 0.4–0.8], consume alcohol (OR 0.3, 95CI 0.2–0.4) or be overweight (OR 0.6, 95CI 0.5–0.8). Survivors from the lowest socioeconomic group were more likely to be current smokers (OR 1.8, 95CI 1.1–3.0) and overweight (OR 1.5, 95CI 1.1–2.1) than those from the highest socioeconomic groups. Additionally, we analysed the impact of chemotherapy on current BMI and found that chemotherapy significantly increased the odds of excess weight by 50% (OR 1.5, 95CI 1.05–2.3, adjusted for the all covariates). Alcohol consumption at the time of the study was also more likely among those who received chemotherapy.

**Table 4:** Demographic and clinical factors that predict current smoking, alcohol consumption and excess weight: multivariate analysis

	Smoking^a^ [OR (95CI)]^b^	Alcohol^a^ [OR (95CI)]^b^	Excess weight^a^ [OR (95CI)]^b^
Gender (ref: male)			
Female	0.5 (0.4–0.8)	0.3 (0.2–0.4)	0.6 (0.5–0.8)
Age (years) at diagnosis (ref: ≥65)			
25–44	3.4 (1.3–9.0)	2.4 (0.9–6.6)	1.2 (0.5–2.6)
45–64	2.5 (1.7–3.8)	1.9 (1.4–2.6)	1.0 (0.7–1.3)
Site (ref: colon)			
Rectum	1.2 (0.8–1.8)	0.7 (0.5–1.0)	0.8 (0.6–1.1)
Stage (ref: stage I)			
II	2.1 (1.3–3.5)	1.0 (0.7–1.4)	1.2 (0.8–1.6)
III	1.3 (0.8–2.3)	0.9 (0.6–1.4)	1.2 (0.8–1.6)
IV	2.3 (1.0–5.2)	0.5 (0.3–1.0)	1.0 (0.6–1.9)
Time (years) since diagnosis (ref: <2 years)			
2–4.9	0.9 (0.5–1.8)	1.0 (0.7–1.6)	1.1 (0.7–1.6)
≥5	0.6 (0.3–1.1)	0.8 (0.5–1.4)	0.9 (0.6–1.4)
SES (ref: III: highest SES)			
I (lowest)	1.8 (1.1–3.0)	0.4 (0.2–0.5)	1.5 (1.1–2.1)
II (middle)	1.5 (1.0–2.3)	0.9 (0.6–1.3)	1.3 (1.0–1.7)
Comorbidity at diagnosis (ref: none)			
Yes	1.1 (0.7–1.6)	0.6 (0.4–0.8)	1.78 (1.4–2.3)
Chemotherapy			
Yes	0.9 (0.5–1.6)	1.7 (1.1–2.7)	1.5 (1.1–2.3)

^a^Lifestyle factors including smoking status, alcohol consumption and BMI were categorised as follows: (1) current smoking: yes or no (includes lifelong non-smokers and ex-smokers), alcohol consumption: yes and no (includes ex-consumer or never consumed alcohol) and (3) excess weight: overweight (BMI 25–29.9 kg/m^2^) and obese (BMI ≥ 30 kg/m^2^)

^b^ *OR (95CI)* odds ratio (95% confidence interval) adjusted for age at diagnosis, gender, site, stage, time since diagnosis, socioeconomic status and comorbidity at diagnosis

## Discussion

In our population-based study, only 8 and 18% of male and female CRC survivors met the advised healthy lifestyle of not smoking, not drinking alcohol and having a normal body weight. Although the prevalence of current smokers and alcohol consumption among survivors was lower as compared to the general Dutch population, more than 28% of the survivors had two or more unhealthy lifestyle factors. Survivors also showed a larger proportion of excess weight as compared to the general population. We found that gender, socioeconomic status and chemotherapy were significantly associated with current opted unhealthy lifestyle or weight in our study.

Similar to our findings, previous studies reported lower prevalence of unhealthy lifestyle factors, namely smoking, among survivors compared to the general population (Table 5) [1, 5, 14, 15]. As compared to the proportion of smokers reported among CRC survivors in most studies in the USA, smoking was less prevalent among US survivors than among Dutch survivors [7]. Prevalence of smoking among the general population is also lower in the USA (21% [16]) than in the Netherlands (27% [13]), suggesting that population-level tobacco policy also influences behaviour of certain group such as cancer survivors. The low prevalence of smoking and alcohol consumption among survivors in our study, even though these lifestyles are accomplished risk factors for CRC [17], might be explained by two causes: firstly unhealthy lifestyles increase mortality either due to CRC or other causes. Thus current survivors may smoke or drink less because some of the smokers or drinkers have died earlier after CRC diagnosis. Secondly, survivors are a group more motivated to change their lifestyle [18], thus the lower prevalence of smoking and alcohol drinking.

**Table 5:** Results for previous studies that have estimated the post diagnosis prevalence of lifestyles and excess weight among CRC survivors

Health behaviours	Present study		Hawkes et al. [24]				Satia et al. [15]				15 studies
	CRC survivors	Population	CRC survivors			Control	CRC survivors		Control		CRC survivors
	*n* = 1349	*n* = ±10000	*n* = 1250			*n* = 6277	*n* = 278		*n* = 459		
			Pre-diagnosis	After 6 months	After 12 months		At diagnosis	After 2 years	Baseline	After 2 years	
	(%)	(%)	(%)	(%)	(%)	(%)	(%)	(%)	(%)	(%)	(%)
Current smokers	11	27	–	7	8	13	–	–	–	–	5 – 22
Excess weight											
Overweight	48	35	39	35	39	40	37	42	40	42	27 – 45
Obese	16	12	27	18	21	20	32	32	33	28	15 – 33
Non-drinker	33	20	–	33	30	37	66	66	67	62	15 – 23

Excess weight seems to be a prominent issue among the survivors. It remained a long-term problem among survivors; in our study BMI did not differ between those diagnosed 6–24 months ago with those diagnosed 24–49 or longer than 50 months before the questionnaire was completed (results not shown). This was observed despite the fact that some cancer survivors can be malnourished and underweight at diagnosis due to advanced disease or as a result of aggressive treatment. Some of these underweight patients might have died early after diagnosis, resulting in an over-representation of longer-term survivors with excess weight [4]. On the other hand, excess weight is a known risk factor for CRC [19]. This might be the reason for the large prevalence of survivors with excess weight in addition to the previously mentioned fact (possible over-representation of survivors with excess weight). This is further compounded by the fact that chemotherapy has been related to weight gain [6]. Current alcohol intake was also associated with a history of chemotherapy. Yet other studies have never reported this and there is not yet any plausible biological explanation behind this finding, so there is currently no reason to think that this result has meaningful impact. On the other hand, we found a 50% higher odds of being overweight even after correcting for age, sex, follow-up time and other possible confounders. A few clinical trials on the use of various chemotherapies, mainly comparing oxalipatin, 5-fluorouracil and folinic acid (FOLFOX), reported that 8–40% of the patients on the therapy gained significant weight [20–22]. Research on the relation between chemotherapy and weight gain among CRC patients was scarce or almost non-existent. For breast cancer, chemotherapy has been related to factors such as (a) changes in resting metabolic rate, (b) thermogenesis, (c) reduced physical activity, and (d) change in dietary habit (more snacking due to nausea related to chemotherapy). One or more of these mechanisms may also play a role in weight gain among CRC patients who received chemotherapy, though research specifically for CRC patients is needed.

Our study showed that among CRC survivors, those who are male and/or belonging to the lowest socioeconomic groups were more likely to have an unhealthy lifestyle. Survivors who had chemotherapy or other illness at diagnosis were also more often overweight. These groups seem to be a good target for intervention programs aimed at improving lifestyle or body weight. The low level of reported physical activity among survivors, also at 12 months since diagnosis [14], suggests a window of opportunity to gain healthy weight through increased physical activity. Furthermore, unhealthy BMI may increase occurrence of comorbid cardiovascular diseases among CRC survivors, thus impairing prognosis [14]. So overall, obtaining a healthy weight through diet and physical activity should be one of the main health messages to be promoted among the survivors.

Our results also showed that 28–33% of the survivors had two or more unhealthy lifestyle factors combined with excess weight. This clustering suggests that a lifestyle intervention targeting multiple behaviours at the same time rather than single behavioural intervention program should be developed for these survivors.

### Strengths and limitations of the study

Our data is based on a large population-based database from a random sample of the survivors with differing years of follow-up, thus ensuring its representativeness of CRC survivors in general in the Netherlands. We did not collect historical (behavioural) data because this might be subject to reporting bias. Currently, a follow-up study is being carried out allowing assessment in lifestyle change and its impact on outcome [23]. As a result of the lack of comparison to cancer patients who did not survive CRC, this study is therefore not meant to identify causal link between lifestyle and survivorship. The findings in this study are based on a cross-sectional survey and should be interpreted with caution. There is likely a survival bias in our sample: those who adopted unhealthy lifestyle were more likely to have died before our study as compared to those who have a healthy lifestyle. Moreover, the elderly and those with comorbidity responded less often to our questionnaire on lifestyle or body weight and height. This may lead to an underestimation of the need for promotion of healthier lifestyle among the CRC survivors in these groups. Finally we took aggregated results of lifestyle and BMI data reported from national survey and used it for comparison to our results. The average age of a sample of CRC survivors from the population is probably higher than a random sample of the general population. Lifestyle is known to differ by age groups, and this may modify comparison between the two groups. Therefore, to overcome this problem, age group comparison was presented in our results.

## Conclusion

Only 8–16% of CRC survivors met all three recommended guidelines of not smoking, not drinking alcohol and having a normal body weight. One of the main health promotion packages for these survivors should include strategies for (re-)gaining healthy weight. Our study calls for multiple behavioural intervention strategies at patient and community level.
